# Future therapeutic strategies in the treatment of extrapulmonary neuroendocrine carcinoma: a review

**DOI:** 10.1177/17588359231156870

**Published:** 2023-03-01

**Authors:** Matthew D. Robinson, Daniel Livesey, Richard A. Hubner, Juan W. Valle, Mairéad G. McNamara

**Affiliations:** Division of Cancer Sciences, School of Medical Sciences, Faculty of Biology Medicine and Health, The University of Manchester, Manchester, UK; The Christie Library, School of Oncology, The Christie NHS Foundation Trust, Manchester, UK; Division of Cancer Sciences, School of Medical Sciences, Faculty of Biology Medicine and Health, The University of Manchester, Manchester, UK; Department of Medical Oncology, ENETS Centre of Excellence, The Christie NHS Foundation Trust, Manchester, UK; Division of Cancer Sciences, School of Medical Sciences, Faculty of Biology Medicine and Health, The University of Manchester, Manchester, UK; Department of Medical Oncology, ENETS Centre of Excellence, The Christie NHS Foundation Trust, Manchester, UK; Division of Cancer Sciences, School of Medical Sciences, Faculty of Biology Medicine and Health, The University of Manchester, Manchester M20 4BX, UK; Department of Medical Oncology, ENETS Centre of Excellence, The Christie NHS Foundation Trust, Wilmslow Road, Manchester, M20 4BX, UK

**Keywords:** extrapulmonary neuroendocrine carcinoma, treatment

## Abstract

Neuroendocrine neoplasms (NENs) are rare malignancies arising most commonly in the gastrointestinal and bronchopulmonary systems. Neuroendocrine carcinomas (NECs) are a subgroup of NENs characterised by aggressive tumour biology, poor differentiation and dismal prognosis. Most NEC primary lesions arise in the pulmonary system. However, a small proportion arise outside of the lung and are termed extrapulmonary (EP)-, poorly differentiated (PD)-NECs. Patients with local or locoregional disease may benefit from surgical excision; however, this is often not an option, due to late presentation. To date, treatment has mirrored that of small-cell lung cancer, with platinum–etoposide forming the basis of first-line treatment. There is a lack of consensus in relation to the most effective second-line treatment option. Low incidence, an absence of representative preclinical models and a lack of understanding of the tumour microenvironment all present challenges to drug development in this disease group. However, progress made in elucidating the mutational landscape of EP-PD-NEC and the observations made in several clinical trials are paving the way towards improving outcomes for these patients. The optimisation and strategic delivery of chemotherapeutic interventions according to tumour characteristics and the utilisation of targeted and immune therapies in clinical studies have yielded mixed results. Targeted therapies that complement specific genetic aberrations are under investigation, including AURKA inhibitors in those with *MYCN* amplifications, BRAF inhibitors in those with *BRAFV600E* mutations and EGFR suppression, and Ataxia Telangiectasia and Rad3-related inhibitors in patients with *ATM* mutations. Immune checkpoint inhibitors (ICIs) have conferred promising results in several clinical trials, particularly with dual ICIs and in combination with targeted therapy or chemotherapy. However, further prospective investigations are required to elucidate the impact of programmed cell death ligand 1 expression, tumour mutational burden and microsatellite instability on response. This review aims to explore the most recent developments in the treatment of EP-PD-NEC and contribute towards the requirement for clinical guidance founded on prospective evidence.

## Background

Neuroendocrine neoplasms (NENs) are a group of malignancies that arise predominantly from tissues of neuroendocrine phenotype in the gastrointestinal and bronchopulmonary systems.^1^ Although NENs are considered rare, with an age-standardised incidence rate of 8.6 per 100,000 in the United Kingdom (UK),^2^ incidence and prevalence are increasing gradually.^3^

**Figure 1.**
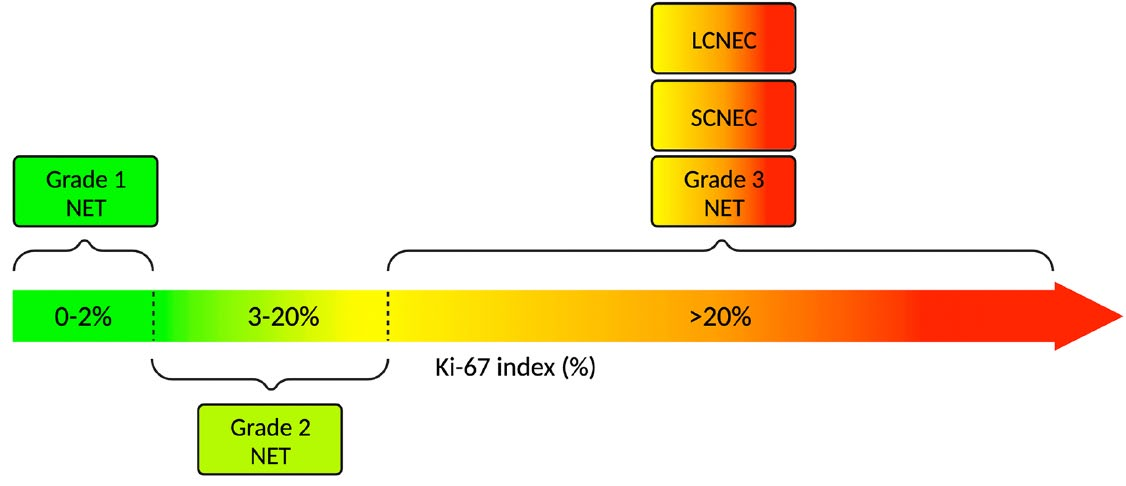
A visual representation of the WHO 2019 classification system for NENs according to Ki-67 index. Source: Based on data from Nagtegaal *et al.*^5^ Created using Biorender.com. LCNEC, large-cell neuroendocrine carcinoma; NEN, neuroendocrine neoplasm; NET, neuroendocrine tumour; SCNEC, small-cell neuroendocrine carcinoma.

NENs are broadly classified based on histopathological evidence of differentiation into two categories: well-differentiated neuroendocrine tumours (NETs) and poorly differentiated (PD) neuroendocrine carcinomas (NECs).^4^ In recent years, a more clinically meaningful classification method, the World Health Organization (WHO) 2010, was established.^4^ This system combines tumour, nodes and metastases staging with descriptive histological criteria based on Ki-67 index to generate three NEN subgroups: Grade 1 (G1), Grade 2 (G2) and Grade 3 (G3), with Ki-67 index criteria of <3%, 3–20% and >20%, respectively. An updated iteration of the WHO classification system published in 2019 separates G3 NENs (previously considered part of the PD group) into well-differentiated G3 NETs and PD-NECs (which present as either small- or large-cell variants) (Figure 1.).^5^ This separation recognises the significant heterogeneity in treatment response and outcomes between the subgroups.^6^ It is thought that G3 NETs are characterised by less aggressive tumour biology, relative to NECs, with NECs exhibiting alterations in tumour protein 53 (*TP53*) and retinoblastoma (*RB1*).^1,7^ The treatment options in G3 NETs differ and an in-depth discussion of these is not included here, as it is beyond the scope of this review.

A significant majority of NEC primary lesions are located in the pulmonary system and are of small-cell origin.^8^ According to a recent comparative analysis based on the National Cancer Institute Surveillance, Epidemiology and End Results database, 8.7% of NECs arise outside of the lung and are termed extrapulmonary (EP)-, PD-NECs.^8^ Approximately 37% of EP-PD-NECs arise in the gastrointestinal system, with more than a quarter originating in an unknown anatomical site.^8^

EP PD-NECs are characterised clinically by an aggressive natural history and poor prognosis, predominantly as a consequence of tumour suppressor gene inactivation. Histopathological characteristics include nuclear atypia, loss of cell architecture, evidence of necrosis and a Ki-67 index of >20%.^5^

## Management and prognosis

Unfortunately, up to 85% of patients present with advanced, unresectable disease with a poor prognosis.^9^ Median overall survival (OS) can be 5.8 months, or less, among these patients.^8^ In a clinicopathological analysis of patients with gastroenteropancreatic (GEP) NECs conducted by Bukhari *et al*., 93% of patients presented with lymph node or distant metastases, a factor that was associated with reduced OS (*p* = 0.0383).^10^

## Resectable cases: Surgery with or without chemotherapy

In contrast, a subset of patients presenting with local or locoregional disease may benefit from surgical excision with curative intent.^11,12^ In these cases, it is essential that occult disease is ruled out with fluorodeoxyglucose positron emission tomography/computed tomography (FDG PET/CT) staging and multidisciplinary discussion is also required.^13^ Many patients will experience recurrence following surgery, with a retrospective analysis of data from 119 patients with pancreatic (Panc)-NECs reporting a median time to recurrence or metastasis of 7 months.^14^ Although the therapeutic role of resection has been observed, the aggressive nature of NEC dictates that adjuvant chemotherapy should be considered in eligible patients.^6^ A National Cancer Database analysis determined that locoregional treatment for GEP-NECs (*N* = 2314) mapped to that of adenocarcinomas at corresponding sites, and that treatment modality, rather than primary site, may determine prognosis.^13^ The authors indicate that colon NECs, anal and oesophageal NECs, and rectal NECs are most likely to receive either surgery and chemotherapy, chemotherapy and radiotherapy, or surgery, chemotherapy and radiotherapy, respectively.^13^ In a retrospective analysis of 806 patients with non-metastatic PD colorectal NECs, adjuvant chemotherapy was associated with a significantly improved median OS time *versus* observation alone (57.4 *versus* 38.2 months, *p* = 0.007).^15^ Notwithstanding the proposed clinical benefit of adjuvant chemotherapy in retrospective studies, there is an absence of randomised controlled trials investigating these interventions. In addition, the role of neoadjuvant treatment is unknown. A phase II trial investigating platinum-based chemotherapy in resectable PD-digestive-NEC is currently ongoing in a non-randomised cohort of 48 participants, with study completion expected in early 2024 (NCT04268121).

## Treatment in the advanced setting

To date, the management of advanced EP-PD-NEC has mirrored the established treatment paradigm of small-cell lung cancer (SCLC). This approach is based on similarities in immunohistochemical characteristics, tumour aggressiveness and morphology. However, evidence of dissimilarity in terms of the genetic drivers of tumorigenesis, disease progression and crucially, response to chemotherapeutic interventions has been demonstrated.^16–18^

Despite this, in advanced cases of EP-PD-NEC, chemotherapy forms the cornerstone of management. Platinum-based combination regimens are recommended as first-line (1L) interventions and are considered standard of care on the basis of clinical benefit in SCLC.^6^ Cisplatin or carboplatin plus etoposide, or carboplatin plus irinotecan are among the most commonly selected options. However, there is no evidence of superiority for any of the established combinations, perhaps as a result of a lack of prospective studies. Interestingly, a recent randomised phase III study of etoposide plus cisplatin *versus* irinotecan plus cisplatin in the 1L advanced setting for patients with digestive NEC (*N* = 170) reported no significant difference in median OS time between the two regimens (12.5 months *versus* 10.9 months respectively, *p* = 0.797).^19^

Although response rates to 1L interventions can exceed 50%, disease control is regularly short-lived [median progression-free survival (PFS) of 5.6 months with cisplatin plus etoposide], generating a need for effective second-line (2L) alternative salvage therapies. There is an area of unmet need in the shape of treatment options beyond 1L chemotherapy and a diverse variety of regimens are used in the 2L setting.^20^ In a systematic review and meta-analysis investigating 2L treatments in 19 studies including 582 patients with EP-PD-NEC, the median response rate (RR) was 18%, and median PFS and OS were 2.5 and 7.64 months, respectively.^20^ These observations underscore the requirement for effective 2L options as a matter of urgency.

## Aims and objectives

This review aims to evaluate the published literature relating to the treatment of EP-PD-NEC in both the 1L and 2L settings to contribute towards the development of an EP-PD-NEC treatment algorithm based on prospective data generated in the prospective setting.

## Methods

A literature search was performed to identify eligible studies and abstracts using the Medline and Embase databases. Keywords used to identify eligible publications included (‘neuroendocrine carcinoma’ OR ‘neuro−endocrine carcinoma’ OR neuroendocrine carcinoma OR ‘EP-PD-NEC’) AND ((‘Treatment’ or ‘Therap*’ or ‘Chemo*’ or ‘SACT’) OR therapy)) AND ((‘grade 3’ or ‘Grade III’ or ‘Grade Three’) OR (‘poorly differentiated’ or ‘High Grade’)) NOT (‘lung’ or ‘well-differentiated’ or ‘well differentiated’ or ‘grade 1’ or ‘Grade I’ or ‘Grade One’ or ‘Grade 2’ or ‘Grade II’ or ‘Grade Two’). No limits relating to date of publication or language were applied.

Eligible studies were required to include results relating to the treatment of PD-NEC originating outside of the lung. Meta-analyses, conference abstracts, prospective studies and retrospective series were included. The references of eligible studies and relevant review articles were examined to identify other relevant studies. Those that were not relevant to the review were excluded on the grounds of relating to well-differentiated NETs, including participants with NEC of pulmonary origin only or relating to Merkel cell carcinoma.

A separate search for relevant ongoing clinical trials was also performed using clinicaltrials.gov. The search term ‘neuroendocrine carcinoma’ was used in the ‘condition or disease’ field and no other terms were applied.

### Results

**Figure 2.**
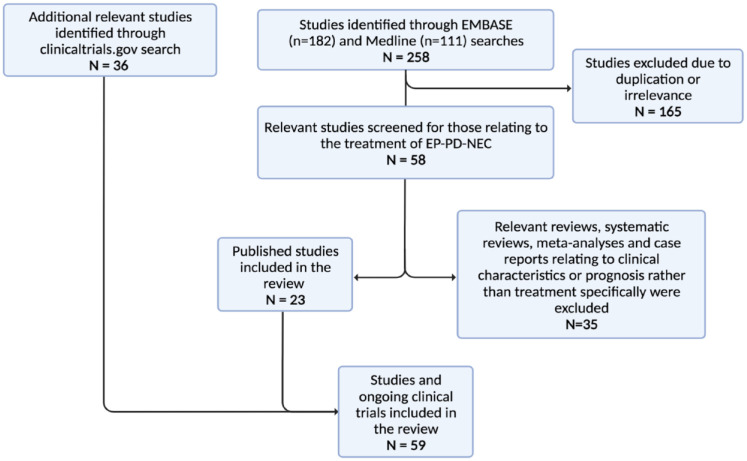
PRISMA diagram indicating the studies and clinical trials included in the review and those that were excluded. Source: Created using Biorender.com. EP-PD-NEC, extrapulmonary poorly differentiated neuroendocrine carcinoma.

The literature search (last updated 18th July 2022) identified 258 publications and 36 additional clinical studies were identified through a search of the clinical trial database (last updated 2nd August 2022). In all, 200 studies were excluded on the basis of duplication, irrelevance and relating to clinical characteristics or prognosis. In all, 23 studies identified through the Embase and Medline database searches, and 36 clinical trials were included in the review (Figure 2). These studies will now be evaluated in greater detail.

## Prospective clinical trials of systemic interventions

Although the response to 1L chemotherapy in patients with advanced NEC is relatively high when compared to those with well-differentiated NETs, outcomes and prognosis remain poor and there is a requirement for more effective chemotherapeutic regimens in the 1L- and 2L settings, biomarkers with the clinical utility to predict response, and a molecular classification system stratified according to chemotherapeutic efficacy. To date, NEC management guidance from international oncologic societies is based primarily on retrospective investigations or therapeutic efficacy in SCLC.^6,21^ However, in recent years, numerous prospective clinical studies have been designed, are recruiting or have reported results, some of which may contribute to the required progress in this area (Table 1).

**Table 1.** Selected studies investigating chemotherapeutic interventions in EP-PD-NEC.

Investigational compound	Trial name	Study design	Site of origin	Line of treatment	Clinicaltrials.gov identifier or alternative	Study status	Estimated/actual enrolment	Recruitment location	Results
Chemotherapy									
5-FU/folinic acid/irinotecan/oxaliplatin or cisplatin or carboplatin + etoposide	FOLFIRINEC	Randomised phase II	GEP-NEC, UNK	1st Line	NCT04325425	Recruiting	218	France	
Capecitabine + temozolomide or cisplatin/carboplatin + etoposide		Phase II	GEP-NEC	1st Line	NCT02595424	Recruiting	126	USA	
Cisplatin + irinotecan followed by octreotide (on completion or progression)	IPO-NEC	Phase II	GEP-NEC	1st Line	NCT01480986	Completed	40	China	
Etoposide + cisplatin *versus* irinotecan + cisplatin	TOPIC-NEC	Randomised phase III	GEP-NEC	1st Line	UMIN000014795	Completed	170	Japan	Results published by Morizane *et al.*,^22^ Median OS = 12.5 months (EP) *versus* 10.9 months (IP), median PFS = 5.6 months (EP) *versus* 5.1 months (IP)
Liposomal irinotecan + carboplatin		Phase I/II	GEP-NEC	1st Line	NCT05385861	Not yet recruiting	52	Taiwan	
Capeciteabine + temozolomide		Phase II	GEP-NEC	Prior systemic therapy allowed	NCT03079440	Active, not recruiting	31	Korea	Results published by Jeong *et al.*,^23^ *n* = 7 (NEC) ORR:14.3%, DCR: 42.9%, median PFS = 3.5 months, median OS = 6.2 months.
FOLFIRI or CAPTEM	SENECA	Randomised phase II	GEP-NEC, Lung NEC	2nd Line	NCT03387592	Recruiting	112	Italy	
Liposomal irinotecan + 5-FU/folinic acid or docetaxel	NET-02	Randomised Phase II	EP-NEC	2nd Line	NCT03837977	Active, not recruiting	102	UK	Preliminary results presented at 2022 ASCO Annual Meeting I by McNamara *et al.*,^24^ (Median follow up = 6.6 mo, *n* = 58): 6 mo PFS rate: 31% *versus* 14.8%, ORR: 10.3 *versus* 10.3%.
Liposomal irinotecan + fluorouracil + leucovorin		Phase II	EP-NEC	2nd Line	NCT03736720	Recruiting	37	USA	
SOX (oxaliplatin/S-1)		Phase II	NEC	2nd Line	NCT03279614	Unknown	45	China	
Temozolomide	TENEC	Phase II	NEC	2nd Line	NCT04122911	Completed	25	Italy	
Temozolomide		Phase II	EP-NEC	2nd Line	UMIN000010549	Completed	13	Japan	Results published by Kobayashi *et al*.,^25^ *N* = 13, RR: 15.4%, median PFS = 1.8 months, median OS = 7.8 months
TAS-102 (trifluridine/tipiracil)	TAS-102 NEC	Phase II	EP-NEC	2nd Line	NCT04042714	Recruiting	14	USA	
TLC388 (Lipotecan)		Phase II	GEP-NEC, SCLC, Thymic-NEC, H&N-NEC, UNK	2nd Line	NCT02457273	Completed	23	Taiwan	Results published by Chen *et al.*,^26^ Median PFS = 1.8 months, median OS = 4.3 months, DCR: 15%, ORR: 0%

CAPOXIRI, capecitabine, oxaliplatin, irinotecan; CAPTEM, capecitabine + temozolomide; DCR, disease control rate; EP, etoposide + cisplatin; EP-NEC, extrapulmonary neuroendocrine carcinoma; EP-PD-NEC, extrapulmonary poorly differentiated neuroendocrine carcinoma; FOLFIRI, folinic acid + 5-fluorouracil + irinotecan; FOLFOX, folinic acid + 5-fluorouracil + oxaliplatin; GEP-NEC, gastroenteropancreatic neuroendocrine carcinoma; H&N, head and neck; IP, irinotecan + cisplatin; mFOLFIRINOX, modified folinic acid + 5-fluorouracil + irinotecan + oxaliplatin; nal-IRI, liposomal irinotecan; ORR, objective response rate; OS, overall survival; PFS, progression-free survival; SCLC, small-cell lung cancer; UK, United Kingdom; UNK, unknown; USA, United States of America; 5-FU, 5-fluorouracil.

In the absence of suitable 2L interventions, especially among patients with deteriorating performance status, aggressive 1L management strategies, or those that incorporate targeted or immune-mediated mechanisms are being explored. These options will be discussed in the following section.

### Treatment in the 1L advanced setting

Platinum-based agents in combination with topoisomerase inhibitors are considered standard of care for 1L treatment in advanced cases of EP-PD-NEC.^6^ There is ambiguity around the potential superiority of one standard of care combination over another, and large-scale, randomised clinical trials are required to address this, although realistically, these may not happen. Alongside the aim to optimise the delivery of existing 1L interventions, several alternative combinations incorporating multiple drug classes are currently being explored (Table 1).

Recent evidence from a randomised phase III trial demonstrated that neither cisplatin plus etoposide nor cisplatin plus irinotecan are superior in terms of efficacy in the 1L management of patients with GEP-NECs.^19^ However, subgroup analyses indicated a potential OS advantage associated with cisplatin plus etoposide in those with NEC of pancreatic origin.^22^ Report of an extraordinary response to platinum-based systemic therapy in a case report of stage IV large-cell NEC of the colon was associated with mutated *BRCA1*, *BAP1* and *BRAF*, high tumour mutational burden (TMB) and microsatellite instability (MSI), supporting the observation that PD disease responds well to platinum-based interventions.^27^ An earlier randomised phase II study also confirmed the non-inferiority of irinotecan plus cisplatin *versus* etoposide plus cisplatin, but the efficacy of irinotecan plus cisplatin was improved in patients with non-small-cell GEP-NEC, albeit non-significantly [objective response rate (ORR) = 30.0% *versus* 14.3%, *p* = 0.42].^28^ In light of the controversy surrounding site-driven *versus* morphology-driven decision-making, further investigations into any potential superiority according to primary site and morphology are required.

The PRODIGE 69-FOLFIRINEC randomised, phase II, prospective trial is investigating the efficacy of 1L oxaliplatin, irinotecan, 5-Fluorouracil (5-FU) and leucovorin (mFOLFIRINOX) combination compared with standard platinum–etoposide chemotherapy (Table 1).^29^ Those patients with grade 3 well-differentiated NETs are excluded and there is stratification according to Ki-67 index prior to randomisation. This aims to facilitate interpretation of efficacy in those with a Ki-67 < 55% *versus* ⩾55% (based on the observation that response to platinum–etoposide chemotherapy is improved in those with Ki-67 > 55%).^30^ The rationale behind using mFOLFIRINOX in NEC is based on retrospective evidence of the clinical efficacy of 5-FU/irinotecan (FOLFIRI) and 5-FU/oxaliplatin (FOLFOX) in the 2L treatment of NEC^31–33^ and in randomised phase II investigations into the 1L treatment of digestive adenocarcinomas.^34,35^ Importantly, PRODIGE 69-FOLFIRINEC will include molecular profiling and targeted next-generation sequencing (NGS) of tumour samples, with the aim to build a profile of actionable molecular drivers of tumorigenesis.^29^

An alternative fluoropyrimidine, capecitabine, in combination with temozolomide (CAPTEM) *versus* platinum-based treatment is under investigation in a randomised phase II study of GEP-NECs in the 1L advanced setting (NCT02595424, Table 1). Interim data from this investigation (*N* = 62) failed to demonstrate superiority of CAPTEM *versus* platinum–etoposide in terms of efficacy, with PFS of 2.43 and 5.36 months, respectively. Although it has been established previously that PD-NECs exhibit an inferior response to CAPTEM when compared to well-differentiated NENs,^23,30^ the efficacy of 1L CAPTEM in PD-NECs relative to platinum-based chemotherapy is not promising based on the above interim data and would not be recommended for patients with PD-NECs based on currently available data.

Prospective evaluation of liposomal irinotecan/carboplatin is planned in a phase I/II single-arm study that will investigate the use of this 1L combination in GEP-NEC, but is yet to recruit (NCT05385861, Table 1).

Progress made in the identification of superior systemic 1L management options in the advanced setting beyond platinum-based systemic treatment is limited, to date. A small number of large-scale phase II studies aiming to provide clarity regarding the role of fluoropyrimidines and temozolomide in this setting may be beneficial (Table 1). However, heterogeneity in response and survival outcomes associated with the current standard of care options according to Ki-67 index, morphology and primary tumour site suggest that further investigations into the benefit of tailoring existing treatment according to these characteristics are required.

### Treatment in the 2L advanced setting

As previously discussed, there is uncertainty around the most effective and appropriate 2L treatment regimens in patients with EP-PD-NEC. This is further complicated by the small proportion of patients that go on to receive a 2L intervention, which at 31% in a recent retrospective study, limits the ability to conduct clinical trials in this population.^36^

Given that many patients will develop resistance to platinum-based chemotherapy, a number of interventions utilising alternative drug classes in the 2L setting are being trialled (Table 1).

NET-02 is a phase II non-comparative trial with 58 participants randomised to receive either liposomal irinotecan (nal-IRI)/5-FU/folinic acid combination or docetaxel as 2L therapy for patients with progressive EP-PD-NEC.^37^ At a median follow-up of 8.1 months, the two interventions were associated with 6-month PFS rates of 31 and 13.8%, respectively, and objective response was achieved in 10.3% of participants in both trial arms^24^ (Table 1). Median PFS was 3 months in those that received nal-IRI/5-FU/folinic acid and 2 months for docetaxel.^24^ Liposomal irinotecan, a topoisomerase I inhibitor, not only plays a role in tumour growth inhibition but is hypothesised, as a result of preclinical investigations, to decrease tumour hypoxia and facilitate the uptake of 5-FU/folinic acid.^38^ The potential role of nal-IRI/5-FU in the 2L advanced setting in combination with additional compounds is also under investigation in GEP-NEC (NCT03736720). A novel, alternative camptothecin analogue, TLC388 Hydrochloride (TLC388 HCl), with structural modifications to improve anti-tumour activity and tolerability, has been developed and demonstrated superior inhibitory activity *versus* topotecan *in vitro*.^39^ Unfortunately, the sparsity of preclinical models emulating the biology of NEC dictates that investigators must proceed with caution when applying preclinical evidence of efficacy to NEC in the clinical setting. The phase II study of TLC388 HCl in the 2L management of NEC illustrates this point, with an ORR of 0%, disease control rate (DCR) of 15% and median PFS of 1.8 months.^26^

In addition to the potential role in the 1L setting, CAPTEM as a 2L intervention in patients with advanced NEC is also being investigated in the SENECA trial; a randomised, phase II study with an active comparator of FOLFIRI (NCT03387592, Table 1).^40^ Temozolomide monotherapy in the 2L treatment of EP-PD-NEC achieved a RR of 15.4% and DCR of 23.1%.^25^ PFS remained modest at 1.8 months and response was not significantly different according to Ki-67 index.^25^ Median OS was 7.8 months in this cohort. However, with a median Ki-67 index of 60% and a recruitment period spanning from 2013 to 2017, it is possible that a proportion of participants may have had G3 well-differentiated NETs. 2L temozolomide was also evaluated in the recently completed phase II, single-arm, TENEC trial in those with advanced NEC of any origin (NCT04122911, Table 1), although results are not yet available.

Trifluridine/tipiracil combination, or TAS-102, is a novel anti-neoplastic intervention without cross-resistance to alternative fluoropyrimidines.^41,42^ Although fluoropyrimidines are not commonly utilised in the 1L management of EP-PD-NEC, several investigations are trialling compounds from the class in this setting. This, considered alongside the demonstrated OS benefit conferred by TAS-102 in metastatic gastric and colorectal cancer,^43,44^ identifies TAS-102 as a reasonable candidate for 2L management of EP-PD-NEC and the compound is under investigation in a phase II trial with completion expected in 2023 (NCT04042714, Table 1).

Given the limited range of effective systemic options available to patients with EP-PD-NEC, targeted strategies aiming to personalise the approach to management are the subject of multiple clinical trials and are discussed in the upcoming section of this manuscript (Tables 2 and 3).

**Table 2.** Selected studies investigating targeted interventions in EP-PD-NEC.

Investigational compound	Trial name	Study design	Site of origin	Line of treatment	Clinicaltrials.gov identifier or alternative	Study status	Estimated/actual enrolment	Recruitment location	Results
Targeted therapy									
Alisertib		Phase II	Prostate-NEC	1st or 2nd Line	NCT01799278	Completed	60	USA	Results published by Beltran *et al.*,^45^ Median follow-up time: 9.7 months, *n* = 45, median PFS = 2.0 months, median OS = 9.5 months, progression-free at 6 months: 16.7%.
Everolimus	EVINEC	Phase II	NEC	2nd Line	NCT02113800	Completed	40	Germany	
Everolimus	NECTOR	Prospective phase II	Panc-NEC	2nd Line		Completed	25	Japan	Results published by Okuyama *et al.*,^46^ Median PFS = 1.2 months, median OS = 7.5 months, ORR: 0%, DCR: 39.1%
Everolimus after partial or complete response to 4–6 cycles of cisplatin/carboplatin + etoposide or alternative *versus* observation		Phase II	GEP-NEC	2nd Line	NCT02687958	Unknown	30	Italy	
PEN-221		Phase I/IIa	EP-NEC	2nd Line or later	NCT02936323	Completed	89	USA, UK	

DCR, disease control rate; EP, extrapulmonary; EP-PD-NEC, extrapulmonary poorly differentiated neuroendocrine carcinoma; GEP-NEC, gastroenteropancreatic neuroendocrine carcinoma; ORR, objective response rate; OS, overall survival; Panc-NEC, pancreatic neuroendocrine carcinoma; PFS, progression-free survival; UK, United Kingdom; UNK, unknown; USA, United States of America.

**Table 3.** Selected studies investigating combination chemotherapeutic and targeted interventions in EP-PD-NEC.

Investigational compound	Trial name	Study design	Site of origin	Line of treatment	ClinicalTrials.gov Identifier or alternative	Study status	Estimated/actual enrollment	Recruitment location	Results
Combination chemotherapy and targeted therapy									
Everolimus + cisplatin		Phase II	EP-NEC	1st Line	NCT02695459	Active, not recruiting	39	Netherlands	Results published by Levy *et al*.,^47^ *n* = 39, DCR: 82.1%, ORR: 58.9%, median duration of response = 6.4 months, median PFS = 6.0 months, median OS = 8.7 months
Everolimus + temozolomide		Phase II	GEP-NEC	1st Line	NCT02248012	Completed	38	Norway	
Elimusertib + irinotecan/topotecan		Phase I	SCLC, NEC, Panc-Ca	2nd Line or later	NCT04514497	Recruiting	96	USA	
FOLFIRI + bevacizumab or FOLFIRI	PRODIGE 41-BEVANEC	Randomised Phase II	GEP-NEC, UNK	2nd Line	NCT02820857	Recruiting	145	France	Results presented by Walter^48^ (*n* = 133) 6-month survival rate = 50.9%, median PFS = 3.7 months *versus* 3.5 months (FOLFIRI alone), median OS = 7.0 months *versus* 8.9 months. ORR: 25.5% *versus* 18.3
Nab-paclitaxel + bevacizumab		Phase II	NEC	2nd Line or later	NCT04705519	Recruiting	100	China	
Lurbinectedin + berzosertib		Phase I/II	SCLC, NEC	Phase I: 2nd Line or later	NCT04802174	Recruiting	75	USA	

DCR, disease control rate; EP-NEC, extrapulmonary neuroendocrine carcinoma; EP-PD-NEC, extrapulmonary poorly differentiated neuroendocrine carcinoma; FOLFIRI, folinic acid + 5-fluorouracil + irinotecan; GEP-NEC, gastroenteropancreatic neuroendocrine carcinoma; NEC, neuroendocrine carcinoma; ORR, objective response rate; OS, overall survival; Panc-NEC, pancreatic neuroendocrine carcinoma; PFS, progression-free survival; SCLC, small-cell lung cancer; UNK, unknown; USA, United States of America.

## Targeted therapies in NEC

### Single-agent interventions

There are a small number of clinical trials investigating targeted agents as monotherapy in advanced EP-PD-NEC (Table 2). Evidence from the literature indicates that patients with PD-NECs may not respond to targeted therapies as well as those with well-differentiated NETs. For instance, everolimus, an inhibitor of mammalian target of rapamycin (mTOR), has demonstrated efficacy and become a standard therapeutic option in patients with unresectable pancreatic well-differentiated NETs.^6,49^ However, limited, small-scale evidence of efficacy in PD-NENs of the pancreas^50^ motivated a phase II, single-arm study of everolimus in the 2L treatment of PD-NENs, which demonstrated disappointing results^46^ (Table 2). Notably, eligibility criteria excluded patients with G1 and 2 NETs but given that recruitment was based on the WHO 2010 classification system, G3 NETs may have been included in the study. In addition, 61% of participants had a Ki-67 index < 55%, and therefore any observations drawn are of limited significance in regard to EP-PD-NEC. Two subsequent phase II trials are currently investigating everolimus monotherapy in the 2L treatment of NEC (either after failure on platinum-based agents or as maintenance therapy) (NCT02687958 and NCT02113800, Table 2). However, NCT02687958 will exclude NECs with Ki-67 > 55% and NCT02113800 included patients with G3 NETs, potentially limiting the applicability of the data from these trials to EP-PD-NEC.

There is evidence to suggest that molecular selection may improve response to targeted therapies in EP-PD-NEC. Aberrant overexpression of the MYC family of proto-oncogenes is a common feature of EP-PD-NEC but is of particular relevance in prostate-PD-NEC.^18^ Amplification of MYCN is observed in up to 52% of patients with prostate-PD-NEC, identifying MYCN as an important driver of tumorigenesis and a potentially targetable susceptibility in this patient population.^18^ Preclinical evidence of dependence on MYCN for tumour maintenance and subsequent inhibition of Aurora A kinase (AURKA) leading to destabilisation of MYCN and suppression of tumour growth, supports an AURKA inhibitor-based approach in prostate-PD-NEC.^51,52^ Phase II investigation of alisertib (an AURKA inhibitor) in participants with metastatic neuroendocrine prostate cancer (NEPC) and castration-resistant prostate cancer yielded modest results (Table 2).^45^ Among those with NEPC, median PFS and OS were 2.0 and 9.5 months, respectively, and 16.7% of patients were progression-free at 6 months.^45^ Interestingly, exceptional responders demonstrated MYCN and/or AURKA overexpression, suggesting that molecular selection should be implemented in future clinical investigations of AURKA inhibition.^45^

The landscape of genetic aberrations in EP-PD-NEC varies according to the primary site.^53^ A genomic analysis of 25 colon NECs observed that *BRAF* was mutated in 28% of samples, with the majority being *BRAF*^V600E^ mutations.^54^ The challenge associated with providing personalised options in many patients with NEC is that many driver mutations are not targetable. However, selective inhibitors of B-Raf including dabrafenib and encorafenib have achieved radiological response in patients with colon NECs.^54^ Further investigation revealed that response can be predicted according to *EGFR* methylation status, which is characteristically high in colon NEC, leading to the *EGFR* repression required to facilitate response to B-Raf inhibition.^54^ Inhibitors of tropomyosin receptor kinases (TRK), specifically larotrectinib, have demonstrated striking anti-tumour activity and clinical response in a phase II basket study of 55 patients with solid tumours harbouring neurotrophic receptor tyrosine kinase (*NTRK*) gene fusions,^55^ and in a recent study including a single case of SCLC.^56^ Although *NTRK* mutations were found in just a single case (8.3%) of Panc-NEC in a genetic analysis of 12 surgically resected specimens, and *NTRK* copy number variations were found in a single case (2%) of cervical small cell NEC in a retrospective analysis of 51 primary NEC specimens,^18^ there may be clinical benefit associated with the participation of patients with *NTRK* fusion-positive EP-PD-NEC in clinical trials of TRK inhibitors (NCT02576431).

## Targeted therapy in combination with chemotherapy

### 1L treatment of advanced EP-PD-NEC

Given the lack of effective 2L treatment strategies in EP-PD-NEC, an area of focus is the identification of superior 1L combinations. Everolimus was combined with cisplatin in a phase II, single-arm clinical trial in 39 participants with EP-PD-NEC (Table 3).^47^ All patients had primary tumours of PD morphology and the median Ki-67 index was 80%. Although a small number (7.7% of the sample population) of Merkel cell carcinoma cases were included, the study provides a valid evaluation of everolimus/cisplatin combination in a relatively well-selected population of EP-PD-NEC cases. With a median PFS of 6.0 months, median OS of 8.7 months, ORR of 58.9% and 3 participants responding to treatment for >12 months, further investigations into this combination that incorporate molecular profiling and NGS may be helpful in identifying a sub-group of patients for whom 1L everolimus/cisplatin yields improved clinical outcomes.^47^ Interestingly, though, comparison of the clinical outcomes associated with everolimus/cisplatin combination^47^ *versus* everolimus monotherapy^46^ indicates that the superior efficacy of everolimus/cisplatin is likely to be a consequence of platinum-based rather than targeted interventions.

Interindividual heterogeneity in response to platinum-based chemotherapy exists among patients with NECs.^30^ Elucidating the factors driving this disparity in response is crucial if outcomes are to be improved. Retrospective evidence demonstrated that patients with gastrointestinal NECs with a Ki-67 < 55% exhibit lower response rates to platinum-based chemotherapy than those with Ki-67 ⩾ 55% (15% *versus* 42%, *p* < 0.001); however, prospective validation is required.^30^ Consequently, alternative chemotherapy agents yielding improved response rates in cases of NEC with Ki-67 < 55% may require investigation (if response rate is considered a good surrogate for efficacy). A phase II investigation of 1L everolimus/temozolomide combination in 38 participants with advanced GEP-NECs (20% < Ki-67 < 55%) is complete and results are awaited (NCT02248012, Table 3).

Preclinical, *in vitro* evidence of the anti-tumour activity of bevacizumab has been demonstrated in a xenograft model of colon NEC.^57^ Tumour growth inhibition of 84% was observed with bevacizumab, supporting an anti-angiogenic approach in the treatment of NEC.^57^ 1L bevacizumab in combination with capecitabine, oxaliplatin and irinotecan (CAPOXIRI-BEV) achieved an ORR and DCR of 47.4% and 78.9%, respectively, in a phase II study of 19 participants with advanced NEC of the small bowel or colon.^58^ Median PFS was 13 months and the median OS was 29 months.^58^ In this study, responders subsequently received pazopanib maintenance therapy alongside CAPOXIRI-BEV. These observations require further investigation in the randomised 1L setting. Bevacizumab combinations have also been utilised in the 2L setting and will be discussed in the following section.

### 2L treatment of advanced EP-PD-NEC

Bevacizumab in combination with capecitabine or 5-FU/streptozocin has conferred promising clinical activity in well-differentiated NETs of the gastrointestinal tract and pancreas and is an established 1L and 2L therapy in metastatic colon cancer.^59–61^ The PRODIGE 41-BEVANEC study was a phase II randomised trial investigating bevacizumab in combination with FOLFIRI for the 2L treatment of EP-PD-NEC. Preliminary results indicate that bevacizumab in combination with FOLFIRI is associated with improved ORR (25.5% *versus* 18.3%) and response duration compared with FOLFIRI alone.^48^ Median PFS was similar with bevacizumab and FOLFIRI, at 3.7 months *versus* 3.5 months for FOLFIRI alone and 50.9% of patients were alive at 6 months in the FOLFIRI plus bevacizumab arm.^48^ Median OS was shorter by 1.9 months in the combination arm *versus* FOLFIRI alone (7 months *versus* 8.9 months, respectively). However, this was a non-comparative study in which both arms met the threshold to be considered ‘active’, so further conclusions cannot be drawn, preserving the uncertainty around the role of bevacizumab in the 2L management of patients with advanced EP-PD-NEC.^48^ The antiangiogenic, bevacizumab, is also under investigation in combination with nab-paclitaxel in the same setting in a phase II trial that is currently recruiting (NCT04705519, Table 3).

An emerging therapeutic avenue under investigation in NEC is the use of a novel class of small molecule kinase inhibitors, Ataxia Telangiectasia and Rad3-related (ATR) inhibitors. The ATR protein plays a crucial role in the DNA damage response, particularly in cancer cells where the G1 checkpoint is often lost due to mutated tumour suppressor genes.^62^ Elimusertib and berzosertib are examples of ATR inhibitors currently under investigation in phase I or early phase II clinical trials in participants with NECs (NCT04514497 and NCT04802174, Table 3). Berzosertib has been investigated previously in combination with gemcitabine, with or without cisplatin, in a phase I study in patients with advanced solid tumours.^63^ Berzosertib/gemcitabine was well tolerated and demonstrated promising signs of preliminary efficacy (8.3% partial response, 60.4% stable disease).^63^ Importantly, though, no cases of NENs or SCLC participated in the aforementioned study. A phase I dose-escalation study of elimusertib in a range of advanced solid tumours demonstrated tolerability and anti-tumour activity in 22 patients, over 54% of whom harboured lesions that were resistant to platinum-based interventions.^64^ In the same study, NGS revealed that all patients exhibiting a partial response to elimusertib-harboured mutant *ATM* or loss of the ATM protein.^64^ Interestingly, in an extensive molecular characterisation study of high-grade GEP-NENs, including 152 NECs, *ATM* copy number alterations were identified in 33% of colonic primaries and 28% of rectal primaries.^65^ These data provide tentative optimism regarding the potential clinical response to ATR inhibitors in a sub-group of patients with NECs, particularly in future investigations where strategic enrichment of the sample population can be performed.

## Immunotherapy

Following recent progress made in the use of immune checkpoint inhibitors (ICIs) in Merkel cell carcinoma, a malignancy exhibiting neuroendocrine differentiation with features similar to that of NEC,^66^ and in SCLC to improve survival outcomes, clinical trials investigating ICIs in isolation or in combination with chemotherapy, targeted therapy or both are underway in EP-PD-NEC.

Developments in the armoury of immunotherapeutic options available, alongside an improved understanding of biomarkers predicting response, are paving the way for a more personalised approach to treatment in NEC. TMB, programmed cell death ligand 1 (PD-L1) expression, MSI and T-cell infiltration are thought to play a role in predicting response to ICIs in multiple cancer types, particularly with anti-programmed death 1/PD-L1 agents.^67^

MSI status occurs at a variable rate in EP-PD-NEC according to the site of primary origin and fluctuates between series. An investigation into the frequency of MSI performed in a cohort of 89 patients with GEP-NECs and mixed adenoneuroendocrine carcinomas found MSI in 12.4% of cases,^68^ and may be influenced by the adenocarcinoma component. A later retrospective cohort study evaluated the clinicopathological and molecular characteristics of 43 G3 NENs, including 29 NECs of GEP or unknown primary origin, and found MSI in 14.3% of cases of gastric or colonic origin.^69^ A pathological review of 40 specimens from patients with cervical NEC demonstrated that the majority are MSI stable and PD-L1 negative.^70^ However, 91% of small-cell cervical NECs tested for PARP-1 showed PARP expression.^70^ The range for TMB in NEC is relatively high at a median of 5.45–5.68 mutations per megabase, and a genomic analysis of primary or metastatic (82.4%) tumour biopsies from 16 patients with a diagnosis of advanced NEC identified four samples with TMB ⩾ 10.^71,72^

It has been reported that PD-NECs are associated with increased expression of PD-L1 when compared to well-differentiated NENs. In a retrospective analysis of 95 surgically resected LCNECs, 74% of samples were positive for PD-L1.^73^ However, a second retrospective analysis of 98 LCNEC biopsy samples observed PD-L1 positivity in only 16%,^74^ demonstrating considerable variation between series, and may be subject to limitations due to differing methods of analysis, defined cut-offs, recency of biopsy tissue obtained, etc. The expression of PD-L1 is thought to indicate the adoption of immune escape mechanisms and PD-L1 positivity is associated with improved efficacy of interventions targeting PD-L1 or PD-1 in certain cancers.^75^

## Immune checkpoint inhibitors

### Anti-PD-1 monotherapy

The use of 2L interventions targeting the PD-L1/PD-1 axis, such as avelumab, pembrolizumab, spartalizumab, nivolumab and toripalimab, in EP-PD-NEC is under investigation in multiple ongoing clinical trials (Table 4).

**Table 4.** Selected studies investigating ICIs in EP-PD-NEC.

Investigational compound	Trial name	Study design	Site of origin	Line of treatment	Clinicaltrials.gov identifier or alternative	Study status	Estimated/actual enrolment	Recruitment location	Results
ICI									
177Lu-Dotatate + nivolumab		Phase II	GEP-NEC, Lung-NEC, UNK	1st and 2nd Line or later	NCT04525638	Recruiting	30	Spain	
Avelumab	NET-001	Phase II	GEP-NEC, Lung-NEC	1st–3rd Lines	NCT03278405	Completed	10	Canada	Results published by Chan *et al.*,^76^ median PFS = 2.0 months, median OS = 5.7 months
AK104 (cadonilimab)		Phase II	Cervical-NEC	2nd or 3rd line	NCT05063916	Recruiting	18	USA	
Avelumab		Phase II	GEP-NEC	2nd Line or later	NCT03147404	Completed	14	Korea	
Avelumab	AveNEC	Phase II	NEC	2nd Line or later	NCT03352934	Completed	60	Germany	Interim results presented by Fottner *et al.*,^77^ at 2019 ASCO Annual Meeting I. *n* = 16 (NEC G3), median follow-up = 16.5 weeks, DCR: 32%, median duration of disease control = 20 weeks, median OS = 4.2 months
Durvalumab + tremelimumab	DUNE	Phase II	GEP-NEC, UNK, NET	2nd Line	NCT03095274	Active, not recruiting	123	Spain	Results presented by Capdevila *et al.*,^78^ at 2020 ESMO Virtual Congress, 9 month OS rate = 36.1%, ORR: 7.2%, median PFS = 2.5 months
Niraparib and dostarlimab		Phase II	SCLC, NEC (excluding prostate)	2nd Line or later	NCT04701307	Recruiting	48	USA	
Nivolumab + ipilimumab		Phase II Basket	NEC	2nd Line or later	NCT02834013	Recruiting	818	USA	Results published by Patel *et al.*,^79^ *n* = 19, ORR: 26%, clinical benefit rate: 36%, 6-month PFS rate 32%, median PFS = 2.0 months, median OS = 8.7 months.
Nivolumab + ipilimumab	GCO-001 NIPINEC	Phase II	GEP-NEC, Lung NEC	2nd or 3rd line	NCT03591731	Active, not recruiting	185	France	Results presented by Walter *et al*.,^48^ ORR: 14.9%, median PFS = 1.9 months, median OS = 5.8 months.
Nivolumab or nivolumab + ipilimumab		Phase II	Genitourinary	Unknown	NCT03333616	Recruiting	100	USA	
Thymoquione + nivolumab + ipilimumab		Pilot	GEP-NEC	2nd Line or later	NCT05262556	Not yet recruiting	10	USA	
PDR001 (spartalizumab)		Phase II	GEP-NEC	2nd Line	NCT02955069	Completed	116	USA, Australia, Austria, Belgium, Canada, France, Germany, Italy, Japan, Netherlands, Spain, UK	
Pembrolizumab		Phase II	GEP-NEC, UNK	2nd Line	NCT02939651	Completed	21	USA	Results published by Vijayvergia *et al.*,^80^ *n* = 29, DCR: 24.1%, median PFS = 8.9 weeks, median OS = 20.4 weeks.
Pembrolizumab	Pembro NEC	Phase II	EP-NEC	2nd Line or later	NCT03190213	Terminated	6	USA	Results published on NIH Clinicaltrials.gov, Clinical benefit rate 16.7 (0.4–64.1), PFS: 42.5 days, OS: 262 days
Toripalimab		Phase Ib	NEC	2nd Line	NCT03167853	Completed	40	China	Results published by Lu *et al.*,^81^ ORR: 20%, DCR: 35%, median DOR = 15.2 months, median PFS = 2.5 months, median OS = 7.8 months.
Sintilimab		Phase I	NEC	2nd Line	NCT02937116	Active, not recruiting	233	China	Results published by Jia *et al.*,^82^ Median follow-up time = 20.7 months, *n* = 17, ORR: 27.8%, median PFS = 2.1 months, median OS = 10.8 months.

DCR, disease control rate; EP-NEC, extrapulmonary neuroendocrine carcinoma; EP-PD-NEC, extrapulmonary poorly differentiated neuroendocrine carcinoma; GEP-NEC, gastroenteropancreatic neuroendocrine carcinoma; ICIs, immune checkpoint inhibitors; NEC, neuroendocrine carcinoma; ORR, objective response rate; OS, overall survival; PFS, progression-free survival; SCLC, small-cell lung cancer; UNK, unknown; USA, United States of America.

The NET-001 study was a prospective phase II trial of avelumab in 10 participants, including 9 with EP-PD-NEC and 1 with SCLC, who had all received 1 or 2 previous lines of systemic therapy (Table 4).^76^ The median Ki-67 index was 82.5%.^76^ Median PFS and OS were 2.0 months and 5.7 months, respectively, and a single participant experienced disease control.^76^ These disappointing outcomes do not support the use of avelumab monotherapy in patients with advanced high-grade NECs and unfortunately, data relating to translational endpoints, including PD-L1 positivity, MSI status and TMB were not included. Interim results from the AVENEC trial, investigating avelumab in patients with G3 NENs (including 16 G3 NECs) following progression to 1L therapy, corroborate the limited anti-tumour activity observed in NET-001, with a median OS of 4.2 months (Table 4).^77^ The DCR was improved, to 32% after 8 weeks and 4 patients experienced stable disease or partial remission at ⩾ 6 months.^77^ Importantly, though, AVENEC included 11 participants with G3 NETs and the median Ki-67 index for the cohort was relatively low, at 60%.^77^ The preliminary results also pooled the clinical outcomes data for G3 NECs and NETs, limiting the relevance of the observations to high-grade NECs specifically. A third phase II study of avelumab in a small population (*n* = 14) of patients with GEP-NECs is completed and results are awaited. Importantly, this investigation incorporates genomic analysis of tumour samples, a factor that may be crucial in identifying responders based on molecular characteristics (NCT03147404, Table 4).

The anti-PD-1 ICI, pembrolizumab, has demonstrated limited efficacy as monotherapy in EP-PD-NEC. In a combined analysis of two phase II investigations, median PFS and OS were 8.9 weeks and 20.4 weeks, respectively (Table 4).^80^ The DCR was 24.1% and the regimen was well tolerated.^80^ The authors analysed outcomes according to PD-L1 expression and observed no significant difference between response or survival in participants with PD-L1-positive *versus* negative tumours.^80^ Notably, 31% of participants had well-differentiated NENs and only 42% had a Ki-67 > 50%, once again limiting translational impact in the context of NEC. A case report of a patient with colorectal NEC treated with pembrolizumab described a complete response (disease-free in excess of 24 months after pembrolizumab treatment).^83^ Immunohistochemistry confirmed that the patient harboured mutated mismatch repair genes and exhibited MSI; both were considered a consequence of Lynch Syndrome.^83^ These observations support the incorporation of MSI testing in the assessment of those with an EP-PD-NEC diagnosis.

Several alternative anti-PD-1 monoclonal antibodies have also been investigated, including toripalimab, which showed evidence of efficacy in the 2L setting in a phase Ib study including 32 patients with locally advanced or metastatic PD-NECs (65.6% had liver metastases), achieving an objective response in 18.7% of participants in the PD-NEC subgroup (Table 4).^81^ Among those patients with tumours identified as PD-L1-positive (⩾1%), ORR improved to 42.9%, significantly higher than PD-L1-negative participants (*p* = 0.034).^81^ High TMB was also associated with improved response (*p* = 0.03).^81^ Objective response to 2L sintilimab was achieved in 27.8% of 18 patients with advanced NEC (including two cases of lung primary origin and one case of mixed adenocarcinoma-NEC) and median PFS and OS were 2.1 months and 10.8 months, respectively.^84^ Although lacking significance, there was an absolute increase in ORR among participants with PD-L1 positive *versus* PD-L1 negative tumours (66.7% *versus* 25.0%).^84^ These observations justify further examination of the potential clinical benefit of anti-PD-1-directed therapies in this patient population, especially those with high TMB and/or PD-L1-positive status.

Aside from dual ICI regimens, which are discussed later on, a number of ICIs are being investigated in combination with chemotherapy (NCT03136055, NCT05058651 and NCT03728361, Table 5). A large, multicentre phase II/III investigation of toripalimab in combination with 5-FU/Simmtecan/l-leucovorin (FOLFSIM), designed to evaluate the safety and efficacy of the intervention in the 2L setting and compare OS outcomes with etoposide plus cisplatin or carboplatin in the 1L setting, is currently recruiting (NCT03992911).

**Table 5.** Selected studies investigating combination chemotherapy and ICI regimens in EP-PD-NEC.

Investigational compound	Trial name	Study design	Site of origin	Line of treatment	Clinicaltrials.gov Identifier or alternative	Study status	Estimated/actual enrolment	Recruitment location	Results
Combination chemotherapy and ICI									
Atezolizumab + cisplatin/carboplatin + etoposide		Phase II/III	EP-NEC	1st Line or after 1 cycle of platinum + etoposide	NCT05058651	Recruiting	189	USA	
Pembrolizumab or Pembrolizumab + paclitaxel/irinotecan/physician’s choice		Pilot	EP-NEC, UNK	2nd Line or later	NCT03136055	Active, not recruiting	36	USA	
FOLFSIM + Toripalimab		Randomised Phase II/III	Bladder-NEC	2nd Line	NCT03992911	Recruiting	336	China	
Nivolumab + temozolomide		Phase II	NEC, SCLC, NET	2nd Line or later	NCT03728361	Active, not recruiting	55	USA	

DCR, disease control rate; EP-NEC, extrapulmonary neuroendocrine carcinoma; EP-PD-NEC, extrapulmonary poorly differentiated neuroendocrine carcinoma; FOLFSIM, 5-fluorouracil/Simmtecan/l-leucovorin; GEP-NEC, gastroenteropancreatic neuroendocrine carcinoma; ICIs, immune checkpoint inhibitors; NEC, neuroendocrine carcinoma; NET, neuroendocrine tumour; ORR, objective response rate; OS, overall survival; PFS, progression-free survival; SCLC, small-cell lung cancer; UNK, unknown; USA, United States of America.

### Dual immune checkpoint inhibition

In reaction to the modest response and clinical outcomes associated with single-agent anti-PD-1/PD-L1 therapy in patients with advanced PD-NEC, strategies with the intention of increasing efficacy are under investigation (Tables 4–6). Combination of anti-PD-1 and anti-CTLA-4 blockade conferred a significant survival advantage compared to single-agent immune checkpoint inhibition in patients with advanced melanoma (hazard ratio = 0.52, *p* < 0.001)^84^ and has shown potential in several phase II studies including NECs (Table 4).

Combined immune checkpoint blockade with ipilimumab and nivolumab has demonstrated efficacy in a cohort of patients with high-grade NENs within a phase II basket study of multiple rare tumours (Table 4).^79^ The cohort consisted of 11 (58%) participants with PD disease and the median Ki-67 index was 80%.^79^ The ORR was 26% and 32% of participants were progression-free at 6 months.^79^ All participants were microsatellite stable and all but one had a TMB < 10 mutations per megabase.^79^ In the absence of correlation with MSI, PD-L1 expression or TMB, further correlative studies exploring the response and efficacy of ICIs in selected patients with NEC are required. Based on the available clinical data, the role of immunotherapy in NEC remains investigational, and therefore immunotherapy may be beneficial in non-selected patients with NEC. Evidence of efficacy in advanced pulmonary- or GEP-NEC in the randomised setting has been presented in interim results of the GCO-001 NIPINEC trial which indicated an improved response to 2L or third-line (3L) ipilimumab/nivolumab combination compared with nivolumab alone (ORR: 14.9% *versus* 7.2%, respectively) (Table 4).^86^ Interestingly, nivolumab in combination with ^177^Lu-Dotatate, a well-established treatment in patients with well-differentiated GEP-NETs with uptake on Ga 68-Dotatate PET/CT imaging, is also under investigation in a sample including PD-NECs (NCT04525638, Table 4). Importantly, it is known that PD-NECs often exhibit decreased Ga 68-Dotatate uptake on imaging and are therefore usually unsuitable for treatments targeting the somatostatin receptor (SSTR).^87,88^ Perhaps in highly selected cases of NEC with high tumour SSTR expression and Ki-67 < 55%, nivolumab/^177^Lu-Dotatate may prove to be a suitable treatment option, but the literature indicates that this is unlikely to extend to a significant proportion of PD-NECs.^89^

Response to another combination of ICIs, durvalumab plus tremelimumab, in 30 patients with G3 GEP-NENs, was limited, at 9.1%, although a 9-month OS rate of 36.1% may justify further evaluation (NCT03095274, Table 4).^78^ Interestingly, PD-L1 expression was not associated with improved response in the cohort of patients with G3 GEP-NENs.^78^ However, neither the number of G3 NETs included in the cohort nor the median Ki-67 index were described in the interim results.

A novel approach to dual inhibition, with a bi-specific anti-PD-1/CTLA-4 inhibitor, cadonilimab, is currently under investigation in the 2L and 3L setting in a phase II study aiming to recruit 18 participants with recurrent or metastatic cervical NEC. The primary endpoint of the study is to establish the 6-month PFS rate among the eligible participants (NCT05063916).

## ICI and targeted therapy combination

As an adjunct to monoclonal antibody-based immune checkpoint inhibition, the therapeutic role of small molecule tyrosine kinase inhibitors (TKIs) in EP-NEC is under investigation (Table 6) with ICIs, and also in triple therapy with chemotherapy and targeted agents (NCT05142865, Table 6). Surufatinib is a TKI acting against VEGFR1-3 and FGFR1 that, combined with toripalimab, has demonstrated promising efficacy and tolerability in both phase I and II studies in the 2L treatment of patients with EP-NEC (Table 6).^85,90^ With an ORR of 23.8% and a DCR of 81% in a phase I study including 13 NECs, further phase II exploration was justified.^85^ Importantly, the phase I study included participants with well-differentiated NETs, including G3 NETs. Similar results were presented in the phase II setting, with an ORR and DCR of 23.8% and 71.4%, respectively.^90^ Median PFS was 4.14 months and median OS was 10.18 months among the 21 participants. A caveat to these promising results is that, although 85.7% of participants had lesions with Ki-67 > 55%, the median value was not presented in the interim results, preventing exclusion of the possibility that G3 NETs were included.

**Table 6.** Selected studies investigating combination ICI, targeted therapy and chemotherapy regimens in EP-PD-NEC.

Investigational compound	Trial name	Study design	Site of origin	Line of treatment	ClinicalTrials.gov Identifier or alternative	Study status	Estimated/Actual enrolment	Recruitment location	Results
Combination ICI and targeted therapy									
Surufatinib or Surufatinib + Toripalimab		Phase II	NEC	2nd Line	NCT04169672	Recruiting	260	China	Results presented by Lu *et al.*,^85^ at NANETS 2021, *n* = 21, ORR: 23.8%, 47.6% of patients (*n* = 10) had stable disease. Six patients (28.6%) had progressive disease, DCR: 71.4% with surufatinib/toripalimab. The median DOR was 4.11 months, median follow-up of 11.07 months, median PFS was 4.14 months, median OS = 10.32 months.
Cabozantinib S-malate + ipilimumab + nivolumab		Phase II	NEC	2nd Line	NCT04079712	Active, not recruiting	30	USA, Canada	
Combination ICI, targeted therapy and chemotherapy									
Camrelizumab + carboplatin/cisplatin + etoposide + apatinib		Phase II	EP-NEC	1st Line	NCT05142865	Not yet recruiting	30	China	

DCR, disease control rate; EP-NEC, extrapulmonary neuroendocrine carcinoma; EP-PD-NEC, extrapulmonary poorly differentiated neuroendocrine carcinoma; ICI, immune checkpoint inhibitor; NEC, neuroendocrine carcinoma; ORR, objective response rate; OS, overall survival; PFS, progression-free survival; SCLC, small-cell lung cancer; USA, United States of America.

### Barriers to progress in the development of therapeutics in this disease group

The challenges associated with developing novel EP-PD-NEC treatment agents, or identifying effective combinations of existing interventions, are multifactorial: low incidence rates may constrain recruitment to clinical trials and therefore limit the range of interventions that can be investigated; drug development is trammelled by the paucity of representative *in vitro* and *in vivo* preclinical models, and a lack of understanding of the tumour microenvironment and mutational landscape of EP-PD-NEC limits therapeutic development.

### Molecular profiling in EP-PD-NEC

Recent progress made in elucidating the mutational landscape of SCLC and the establishment of molecular subtypes based on genomic and transcriptomic profiling studies has revealed targetable vulnerabilities in *TP53*, *RB1*, *KIAA1211*, *COL22A1* and *NOTCH* family genes.^91^ Whilst this is a positive step forward in personalised medicine, these investigations have highlighted an area of unmet need in EP-PD-NEC. In response, investigations aiming to explain the genomic alterations driving EP-PD-NEC have been conducted, albeit in small sample sizes, often restricted to single primary sites, with comprehensive genomic profiling seldom performed.^53,65^ In a genomic analysis of 135 high-grade EP-PD-NEC, *TP53*, *KRAS*, *APC* and *ARID1A* were mutated in 51%, 30%, 27% and 23% of primary tumours, respectively.^53^ These observations were corroborated by a subsequent analysis of tumour samples collected either following resection (32.9%) or biopsy from 152 patients with EP-PD-NEC (79.6% of which had metastatic disease), with the addition of mutant *BRAF* in 20% of samples, and amplifications in *MYC* (51%) and *KDM5A* (45%).^65^ The aforementioned studies included samples from patients with GEP-NECs only. In a recent genomic profiling study performed by Yachida *et al.*,^7^ two subgroups of pancreatic NECs (Panc-NECs) were established: ‘acinar-type’ Panc-NECs, characterised by *TP53* alterations and intact *RB1*; and ‘ductal-type’ Panc-NECs, displaying inactivated *TP53* and *RB1* and overexpression of neuroendocrine transcription factors. The authors also observed distinct genomic differences between Panc-NECs and non-Panc-NECs.^7^ Non-Panc-NECs were more commonly associated with nonsynonymous mutations (*p* = 0.00238), Notch family gene mutations and structural variants than Panc-NECs.^7^ The authors also observed that 5-year disease-specific survival was significantly poorer in those with Panc-NECs than non-Panc-NECs (*p* = 0.0382).^7^

#### Preclinical models of EP-PD-NEC

The scarcity of preclinical models that accurately recapitulate the tumour biology of EP-PD-NEC is the source of a significant barrier to the development of novel therapeutic approaches. The development of histopathologically representative genetically engineered mouse models has been instrumental in facilitating preclinical and translational research in SCLC.^92,93^ The relative shortfall in the development of EP-PD-NEC models is reflected in the dismal prognosis associated with these malignancies and the sparsity of effective 2L interventions.

There is emerging evidence supporting the use of circulating tumour cells (CTCs) to generate CTC-Derived eXplants in SCLC that mirror donor treatment response *in vivo*.^94^ The use of CTCs and cell-free DNA to generate similar mouse models in EP-PD-NEC is currently under investigation. Interestingly, the recent development of a CTC-Derived eXplant model of Merkel cell carcinoma that mirrors donor tumour biology and treatment response is a crucial step towards the use of patient-relevant models in preclinical studies and potentially in guiding future clinical decision-making for patients.^95^

## Discussion

The studies included in this review are heterogeneous in many respects. Clinical trial sample sizes vary considerably between studies, ranging from 6 to 818 participants. Several studies restrict eligibility according to primary site and others recruit participants with NEC from any (including pulmonary) or unknown origin. The evolving classification system for NENs over the past decade dictates that the eligibility criteria of earlier (pre-2017) studies facilitate the inclusion of well-differentiated G3 NETs, limiting the applicability of the results to PD-NEC. Although it is necessary for future clinical trials to recruit internationally, the included studies were conducted across four continents, drawing attention to the potential impact of geographical heterogeneity among individuals with EP-PD-NEC.

Although translational endpoints are being incorporated into clinical trials more regularly, several investigations omit data that are crucial in interpreting responses to interventions based on pathological characteristics, genetic aberrations or the immune microenvironment.

To address the challenges raised in this review, and in light of the evidence presented herein regarding future treatments for EP-PD-NEC, several recommendations can be made in relation to the design of forthcoming clinical studies, priorities for preclinical investigations and patient management. These will be detailed in the following section.

### Future directions and recommendations

There is a requirement for large, international, multi-centre prospective clinical trials in EP-PD-NEC. As a consequence of the rarity of the disease, the majority of previous and ongoing clinical trials are conducted in small sample populations, limiting the statistical power and clinical applicability of the observations made. Widening the geographical recruitment area leads to an increase in the population of eligible participants. Alongside this, basket studies can be designed to investigate the safety and efficacy of interventions in a larger population of participants with multiple cancer types and the inclusion of patients with EP-PD-NEC in these studies may be beneficial.

To exploit the continually evolving progress made in elucidating the mutational landscape in EP-PD-NEC, the findings of molecular profiling studies should inform the design of more efficacious clinical trials with study samples enriched for selected molecular or histopathological characteristics. In addition, future prospective clinical trials that incorporate translational correlates may inform the planning and execution of biomarker-driven studies.

The development of *in vivo* models that accurately recapitulate the biology of EP-PD-NEC is essential for the development of novel interventions and the subsequent design of clinical trials that adopt the findings of preclinical investigations.

### Management recommendations

**Figure 3.**
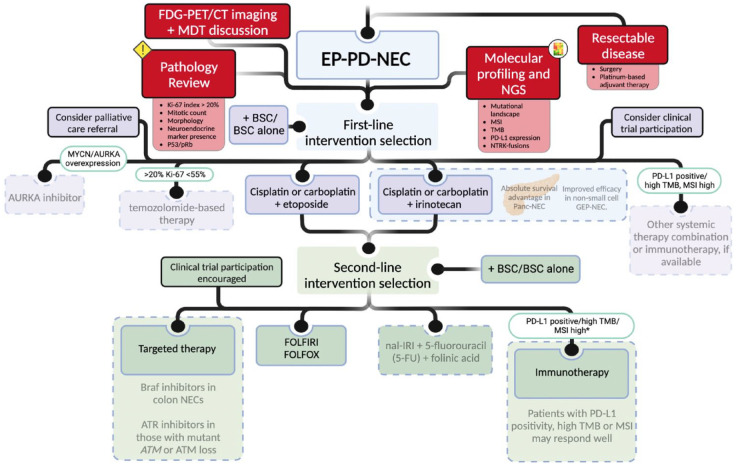
Recommended diagnostic and management pathways for patients with advanced EP-PD-NEC. Explanations contained within the dotted boxes indicate data that require further prospective studies in order to be more definitive in recommending these specific therapeutic options. *In patients that are immunotherapy naïve. Source: Created using Biorender.com. ATR, ataxia telangiectasia and Rad3 related; AURKA, aurora kinase A; BSC, best supportive care; CAPOXIRI, capecitabine, oxaliplatin, irinotecan; CAPTEM, capecitabine + temozolomide; EP-PD-NEC, extrapulmonary poorly differentiated neuroendocrine carcinoma; FDG PET/CT, fluorodeoxyglucose positron emission tomography/computed tomography; FOLFIRI, folinic acid + fluorouracil + irinotecan; FOLFOX, folinic acid + fluorouracil + oxaliplatin; GEP, gastroenteropancreatic; MDT, multidisciplinary team; mFOLFIRINOX, modified folinic acid + fluorouracil + irinotecan + oxaliplatin; MSI, microsatellite instability; nal-IRI, liposomal irinotecan; NEC, neuroendocrine carcinoma; NGS, next-generation sequencing; Panc, pancreas; PD-L1, programmed cell death ligand 1; TMB, tumour mutational burden; 5-FU, fluorouracil.

For all patients with a diagnosis of PD-EP-NEC, a pathological review of the tissue sampled from the primary or metastatic tumour site should be performed (preferably by an experienced pathologist in a NET centre of excellence). Following this, the assessment of disease extent *via* FDG-PET/CT imaging and multidisciplinary team discussion is required to determine potential eligibility for surgical intervention. If available, molecular profiling and NGS should be performed with emphasis placed on evaluation for mutant *BRAF* or *ATM, NTRK* gene fusions, MSI, PD-L1 expression and TMB, where therapeutic options may be available (Figure 3). Given the poor prognosis for patients with advanced disease, early palliative care involvement is imperative. Clinical trial enrolment should always remain a consideration. Platinum-based treatments are favoured in the 1L advanced setting, with 5-FU-based therapies being a 2L option (Figure 3).

## Conclusion

Although a diagnosis of EP-PD-NEC is rare, poor prognosis and significant mortality have positioned the malignancy as a priority for research groups investigating novel interventions. Recent progress has begun demystifying the genetic landscape of EP-PD-NEC and has led to mutation-based classification in pancreatic NECs. Crucially, the latest clinical trials have begun to integrate this into their design by incorporating translational endpoints to inform future studies. The next steps should possibly consider stratifying clinical trial participants according to specific drivers of tumorigenesis to maximise response. Combination regimens including ICIs, targeted therapies and chemotherapy may be at the forefront of the evolving treatment armoury and the publication of mature clinical trial data related to these interventions is eagerly awaited.

Although uncertainty remains around the future treatment options for patients with EP-PD-NEC, this review presents reassuring evidence of progress in the development of some preclinical models, novel therapies and prospective clinical studies that may contribute towards improved clinical outcomes in patients with this frequently devastating disease.

## Declarations

**Ethics approval and consent to participate:** Not applicable.

**Consent for publication:** Not applicable.

**Availability of data and material:** Not applicable.
